# Supplementary material for: Efficient prediction of attosecond two-colour pulses from an X-ray free-electron laser with machine learning
Source: Sci Rep. 2024 Mar 27;14:7267. doi: 10.1038/s41598-024-56782-z (PMC10973530; doi:10.1038/s41598-024-56782-z)
Supplement: Supplementary file 1 — Supplementary Information. [file 41598_2024_56782_MOESM1_ESM.pdf]

Supplemental Material: Efficient prediction of attosecond two-colour pulses from an X-ray free-electron laser with machine learning

Karim K. Alaa El-Din[1,*], Oliver G. Alexander[1], Leszek J. Frasinski[1], Florian Mintert[1,3], Zhaoheng Guo[4], Joseph Duris[4], Zhen Zhang[4], David B. Cesar[4], Paris Franz[4], Taran Driver[4], Peter Walter[4], James P. Cryan[4], Agostino Marinelli[4], Jon P. Marangos[1], and Rick Mukherjee[1,2]**

[1]Blackett Laboratory, Imperial College London, SW7 2AZ, London, UK
[2]Center for Optical Quantum Technologies, Department of Physics, University of Hamburg, Luruper Chaussee 149, 22761 Hamburg, Germany
[3]Helmholtz-Zentrum Dresden-Rossendorf, Bautzner Landstraße 400, 01328 Dresden, Germany
[4]SLAC National Accelerator Laboratory, Menlo Park, California 94025, USA
[*]karim.alaael-din@physics.ox.ac.uk
[**]rick.mukherjee@physnet.uni-hamburg.de

Here we present the results of our ML prediction for central photon energies using the experimental setup [1] described in the main text and compare it against the predictions of energies and time delay using data obtained from another two-colour experiment [2] which has a different modus operandi than [1]. Although [2] does not utilize an enhanced SASE scheme like in [1], both methods use a variable line spectrometer to measure the X-ray spectrum. To create the two pulses, a double slotted foil in inserted into the bunch compressor. In the bunch compressor, there is a space-to-energy mapping, so the spatial windows spoil the bunch except in two energy regions, which are then the only regions able to lase. As energy maps to time in the undulators, this space-to-energy mapping becomes a space-to-time mapping for the emission. The result is reduced total brightness and emission confined to two short periods, i.e. pulses. The widths of the slits determine the widths of the pulses, and the slits' separation scales linearly with the delay [3]. The space-to-energy mapping in the bunch compressor is equally important, and will jitter with the electron beam energy. Pulses up to 30 μJ were produced in this way with photon energy centred close to 540 eV and separated by 14 eV. The repetition rate was 120 Hz, though complete pulse diagnostics operated at only 60 Hz. The temporal structure of the pulses is retrieved for the double slotted foil method using XTCAV.

In general, we find higher input-output correlation for the data from [2]. Testing on the highly correlated data with limited non-linearity helps to benchmark our theoretical prediction models.

1 Predicting central photon energies with two-pulse data from experiment in [1]

Figure S1 shows the validity of predicting central photon energies of the individual pulses (E_1 and E_2) using different machine learning methods. Despite the complex inter-dependence of these energies on the diagnostics, which is in some cases highly non-linear, the linear regression model (LIN) makes reasonable predictions of the central photon energy for either pulse, as seen in Fig. S1(a) and (d).
Both gradient boosting (GB) and artificial neural networks (ANN) make better predictions than the LIN models for the central photon energies of the individual pulses as depicted in Fig. S1(b), (e) and Fig. S1(c), (f) respectively. In general, independent of the prediction model, the mean absolute error for the predictions of E_2 are 2.4 times larger than for E_1 which can be attributed to the fact that Pulse 2 is the second harmonic of Pulse 1. Thus, the second pulse will experience effects of electron bunch energy jitter twice as much compared to Pulse 1 while the remaining difference may be attributed to the error in our energy measurements. It is promising to find that the GB model and the ANN model have similar accuracy in their predictions, especially since we find the GB models are faster to train compared to ANN models, at least by a factor of three.

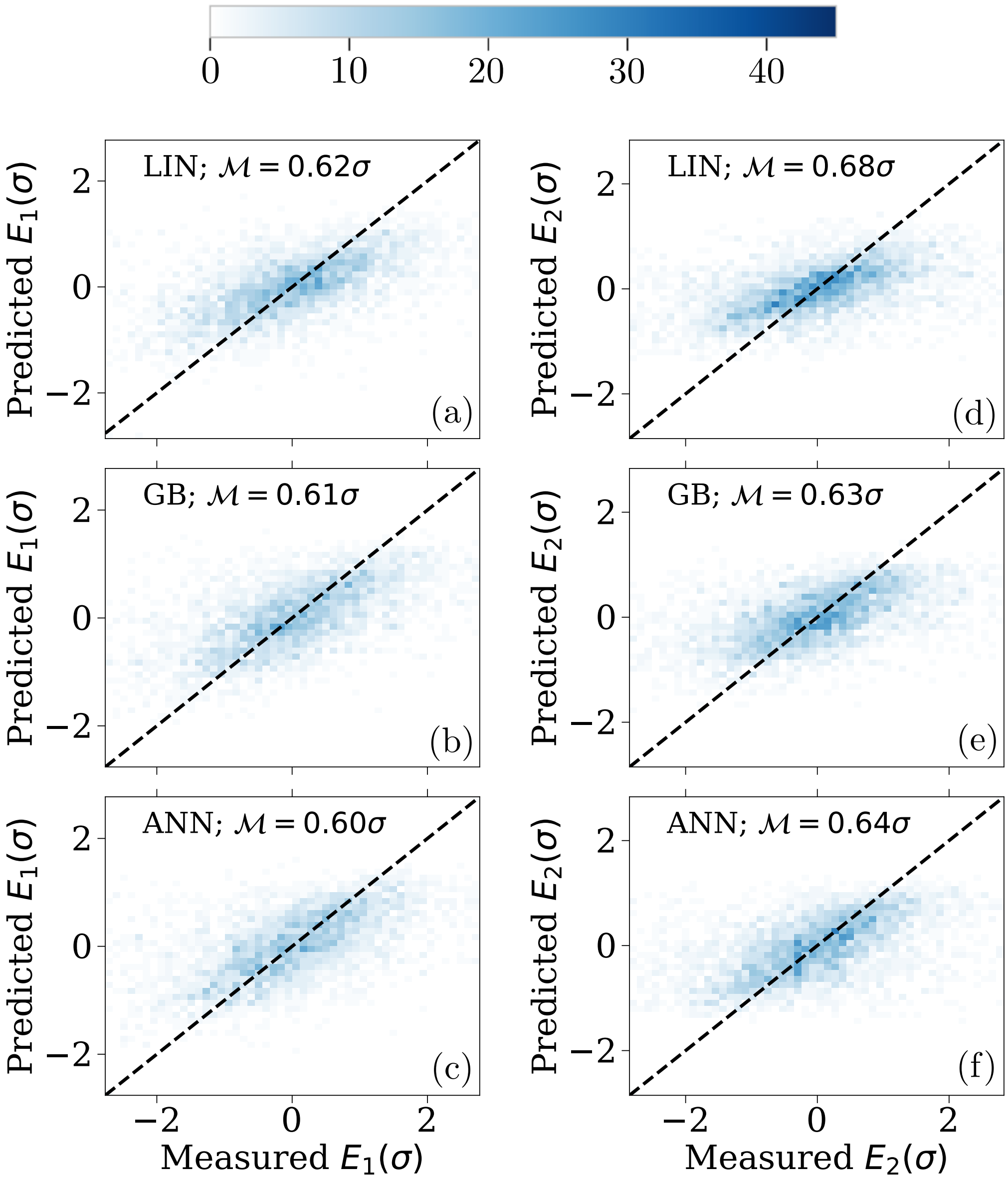

Figure S1. Prediction of XFEL energies of the individual pulses for two-colour data: (a–c) compare the measured values of energies E_1 with the values predicted by different ML methods, while (d–f) is the same for E_2. Top row panels represent linear regression model (LIN), middle row panels represent gradient boosting method (GB) and bottom row panels represent neural networks (ANN). The 2D histogram plots are constructed by grouping the data into 50 bins along each direction, where the density is indicated by the intensity of the blue colouring.

2 Predictions of central photon energy and time delay for two-colour data from experiment in [2]

Figures S2 and S3 depicts the prediction of the central photon energy of the second pulse and time delay between the two pulses using the machine learning methods as used in the main manuscript. While the results agree with [2], it is more efficient in training time due to reduced input parameter space, which is the result of our feature analysis. Interestingly, for this data, the linear model here is sufficient to make accurate predictions with mean absolute error that is a fraction of the variance of the data. This perhaps can be understood by considering how the double-slotted foil affects the electron bunch used to create the pulses. In summary, the data in [2] seems to be less nonlinear in nature with high input-output correlation compared to [1].

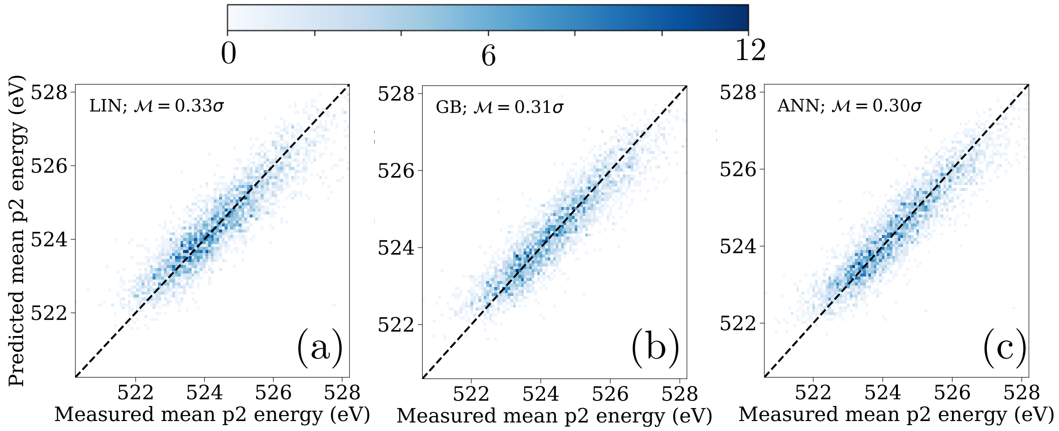

Figure S2. Comparing the measured values of the higher energy pulse from the two-colour data of an older operation mode [2] using the prediction from different ML methods: (a) linear regression model (LIN), (b) gradient boosting method (GB) and (c) neural networks (ANN). 2D histogram plots are constructed in similar way as Fig. S1. Note that the energies shown here differ from those presented in [2] by a small offset owing to a scaling factor but doesn't affect the performance of the fit.

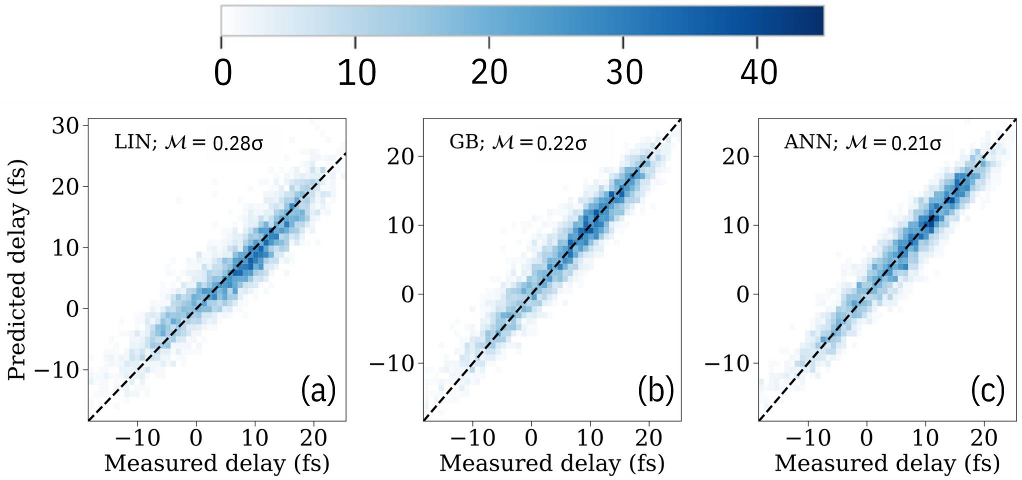

Figure S3. Comparing the measured values of time delay for the two-colour data [2] using the prediction from different ML methods: (a) linear regression model (LIN), (b) gradient boosting method (GB) and (c) neural networks (ANN). The $\mathscr{M}$ values correspond to 2.39 fs, 1.87 fs, and 1.77 fs for LIN, GB, and ANN, respectively.

References

1. Duris, J. *et al.* Tunable isolated attosecond x-ray pulses with gigawatt peak power from a free-electron laser. *Nat. Photonics* **14**, 30–36 (2020).
2. Sanchez-Gonzalez, A. *et al.* Accurate prediction of x-ray pulse properties from a free-electron laser using machine learning. *Nat. Commun.* **8**, 15461 (2017).
3. Ding, Y. *et al.* Generating femtosecond X-ray pulses using an emittance-spoiling foil in free-electron lasers. *Appl. Phys. Lett.* **107**, 191104 (2015).
